# Co-dependency of exchanged behaviors is a cue for agency attribution in 10-month-olds

**DOI:** 10.1038/s41598-021-97811-5

**Published:** 2021-09-14

**Authors:** Tibor Tauzin, György Gergely

**Affiliations:** Department of Cognitive Science, Central European University, Október 6. u. 7, Budapest, 1051 Hungary

**Keywords:** Psychology, Human behaviour

## Abstract

Goal-directed social interactions (whether instrumental or communicative) involve co-dependent, partially predictable actions of interacting agents as social goals cannot be achieved by continuously exchanging the same, perfectly predictable, or completely random behaviors. We investigated whether 10-month-olds are sensitive to the co-dependence and degree of predictability in an interactive context where unfamiliar entities exchanged either perfectly predictable (identical), partially predictable (co-dependent), or non-predictable (random) signal sequences. We found that when—following the interactive exchanges—one of the entities turned in the direction of one of two lateral target objects, infants looked more at the indicated referent, but only in the partially predictable signals condition. This shows that infants attributed agency to the orienting entity and interpreted its turning action as a referential object-directed action. The present findings suggest that the co-dependency and partial predictability of exchanged behaviors can serve as an abstract structural cue to attribute intentional agency and recognize goal-directed social interactions.

Humans as well as several other non-human species (e.g., Ref.^1^) share the basic cognitive adaptation to categorize physical entities as belonging to two ontologically separate domains representing them either as inanimate objects or as animate agents. Young human infants show a remarkable ability to identify intentional agents by relying on their sensitivity to a variety of diagnostic cues that indicate agency. First, infants can recognize that intentional agents possess internal and renewable source of causal energy, which enables them to autonomously generate their own actions^2,3^. Second, they expect intentional agents to possess perceptual, attentional, and representational capacities to detect and encode relevant information from their environment^4,5^. Third, young human infants assume that intentional agents use their perceptual and motor capacities to act in a goal-directed manner optimizing the efficiency of realizing their goals given the constraints that their environmental context impose on possible actions^6,7^. Fourth, infants represent intentional agents as possessing the ability to produce a variety of different actions that allow them to pursue efficient solutions to achieve a given goal in a particular context^8^. These results suggest that infants expect the goal-directed actions of intentional agents to be efficient and predictable—given the agent’s dispositions, intended goals and the environmental constraints—and at the same time to be able to vary their behavior to allow flexible adjustment of their actions to the changes in the situational context. Accordingly, neither completely predictable, invariant responses repeatedly performed in an unmodified manner nor fully variable, but random and non-predictable actions would be efficient to achieve a goal when the situational context is changing. For instance, if a predator would perfectly mirror the behavior of its prey, it would be impossible for the predator to decrease the distance from the prey as both would move in the same direction with the same speed. On the other hand, if the predator and the prey would act randomly the distance between them would vary randomly since the speed and direction of the prey would not affect the speed and direction of the predator.

The degree of predictability of contingently exchanged co-dependent actions during social interactions—in contrast to the predictability of instrumental goal-directed actions based on expected efficiency—might also provide a cue for infants to recognize intentional agents. However, the cues of agency provided by the pattern of interactive actions have not been sufficiently investigated previously. Interacting entities may collaborate to achieve shared goals (e.g., to catch a prey by coordinating their movements or to share information with each other), however, neither of these social aims can be realized by exchanging perfectly repetitive (fully predictable) or completely random (non-predictable) actions. The distance of randomly acting predators, for instance, would vary randomly from the prey and from each other, while the distance of predators imitating each other could not ensure decreasing their distance from the prey. We assume, therefore, that partial predictability and co-dependency of interactive exchanges is a necessary precondition for coordination of individual actions that enable the realization of social goals. Thus, the coordination of actions can provide a diagnostic cue to recognize goal-directed social interactions and to attribute intentional agency to the interactive participants. In the present work, relying on previous results about contingent interactions in infants we shall investigate whether co-dependency and the degree of predictability of exchanged unfamiliar signal sequences by unfamiliar entities can facilitate agency attribution in 10-month-olds.

Early findings revealed that young human infants recognize the basic temporal structure of social interactions and can attribute agency to unfamiliar entities based on their temporally contingent reactivity at a distance alone. In a seminal study, Watson^9^ demonstrated that two-month-olds can discover if their spontaneously produced head movements exert contingent control over the reactions of a toy hanging above their crib. Having detected that their responses controlled the hanging toy’s reactive behaviors from a distance, infants started to produce significantly more head movements. Importantly, the detection of contingent control over the distal object’s behaviors also induced social responses in the infants, such as cooing and smiling at the reactive object. No comparable reactions were induced, however, in a non-contingent yoked-control condition. These results suggest that young infants attribute agency to contingently reacting entities even if they are unfamiliar to them.

Subsequent studies demonstrated that the actions of contingently interacting agents can elicit a referential interpretation of their subsequent object-directed actions in young infants. In several gaze-following experiments an unfamiliar entity, for instance a robot without human-like facial features^10,11^ or a novel, furry entity^5^ produced temporally contingent reactions to the infants’ spontaneous vocalizations and movements. Then, in the test phase the contingently reactive entity performed a 45-degree turning movement so that its frontal part became spatially oriented towards one of two distal target objects positioned at either side of its body. This led infants to gaze-follow the direction of the reactive entity’s turning response and to look longer at the referent object that its orienting response indicated than at the alternative non-target object. In contrast, in the non-contingent yoked control condition—where during familiarization the entity’s actions were independent from the infants’ behavior—there was no comparable gaze-following reaction induced in the infants. This contingency-based gaze-following paradigm was applied in a variety of follow-up studies, which replicated the original findings (e.g., Refs.^4,11–14^). These results with preverbal infants were commonly interpreted as suggesting contingency-based agency attribution to unfamiliar, reactive entities on the one hand and a referential interpretation of their subsequent orienting actions towards distal target objects on the other.

Recently, we found^15,16^ that apart from sensitivity to temporal contingency, that is, *when* a reactive behavior is produced by a potential agent, young infants are also sensitive to *what* reactions are exchanged in turn-taking interactions. In a third-person paradigm infants watched animated videos during familiarization in which two unfamiliar and stationary entities exchanged triplets of non-speech sounds (melodic tones or Morse code beeps) in a temporally perfectly contingent manner. In the Variable Signals condition, the sequence of signals produced by the two entities in each turn contained some amount of variability (e.g., the reaction to A–B–C was A–D–E). In contrast, in the Identical Signals condition, the exchanged signal sequences were always perfectly identical (e.g., the reaction to A–B–C was always A–B–C). Based on the information theoretical insight that the transfer of new information necessarily requires some degree of unpredictability in the signals transmitted^17^, it was hypothesized that the exchange of variable signals—in contrast to fully predictable, identical signals—may serve as a cue for infants to diagnose that communicative information transmission may have taken place between intentional agents.

To investigate this hypothesis, we used the gaze-following paradigm described above with 10-month-olds^16^. We found that in the Variable Signals condition infants looked longer at the target object that the entity oriented towards than at the alternative non-target object on the opposite side. No comparable orientation-following response was induced, however, in the Identical Signals condition or when a Single Entity produced variable signals alone. These results suggest that preverbal infants can represent the exchange of variable signals by two entities—as opposed to exchanging identical signals—as potentially involving communicative information transmission. This induced 10-month-olds to attribute agency to the information transmitting interacting entities and interpret their turning action towards a distal target referentially.

Since communicative signal exchanges are social interactions, we conjecture that infants possess a sensitivity to the *degree* of mutual predictability of actions in interactive contexts when attributing agency. Previous studies support that humans are adapted to recognize and engage in turn-taking contingent social interactions from early on^18–21^, and this special sensitivity appears to be universally present across cultures^22^. Crucially, these social interactions do not normally involve completely unpredictable and independent action sequences produced by the participants. To jointly achieve a social goal, it typically requires mutual coordination and appropriate modification of the complementary actions performed by collaborating agents both in the domain of joint cooperative pursuit of a shared instrumental goal and in the domain of communicative information exchange^23^.

Therefore, we assume that social interactions are in general characterized by medium–high predictability and partial co-dependence of the contingently reactive responses of the collaborating participants to achieve their social goal. Accordingly, observing interacting entities that exchange partially co-dependent signal sequences with medium–high predictability can provide a diagnostic cue indicating that the interacting entities are (1) goal-directed intentional agents, (2) capable of distal perception and attention to each other’s actions and (3) having voluntary motor control over and capacity to reactively modify their own actions to coordinate them with the reactions of their partner.

The previously outlined findings of Tauzin and Gergely^16^, however, did not test whether infants’ sensitivity to predictability of interactive exchanges allows them to monitor for and differentiate between certain levels of predictability when ascribing agency to unfamiliar entities. Moreover, it also left open the question whether infants are adapted to recognize the presence of co-dependence and partial predictability of signals, which characterizes social interactions and communicative information exchange between cooperating agents. If infants are adapted to detect communicative agents who cooperate by mutually coordinating their behavior, they should not attribute intentional agency to interacting entities, which exchange completely unpredictable, random signal sequences that would not satisfy the requirement of partial predictability and co-dependence between the exchanged actions. Therefore, in the present study, relying on the design and results of Tauzin and Gergely^16^ we shall test a crucial condition, which involves exchanges of non-predictable random responses. This will allow us to examine the ability of 10-month-olds to attribute agency to novel entities that exchange unfamiliar signal sequences with (1) full, (2) mid-high or (3) no predictability and co-dependence in the Identical, Variable and Unpredictable Signals conditions, respectively.

We hypothesize that infants will attribute agency to an entity and gaze-follow its subsequent orientating action only when the entity was previously engaged in the exchange of partially predictable and co-dependent—as opposed to fully or non-predictable—signal sequences with its partner. This hypothesis predicts an inverted U-shaped-like function (see Fig. 1c) when the amount of orientation-following gaze responses of infants to look at the target referent indicated by the entity’s turning orientation is plotted against the degree of predictability of the exchanged signal sequences. We also assume that since it is the abstract structural property of the degree of mutual predictability of the exchanged signals that provides a diagnostic cue for agency attribution it will be identifiable irrespective of the specific sound signals realizing it. Therefore, we hypothesize that that the auditory characteristics of the exchanged unfamiliar sound signals—either being Morse codes or melodic tones—will not affect agency attribution.

**Figure 1:**
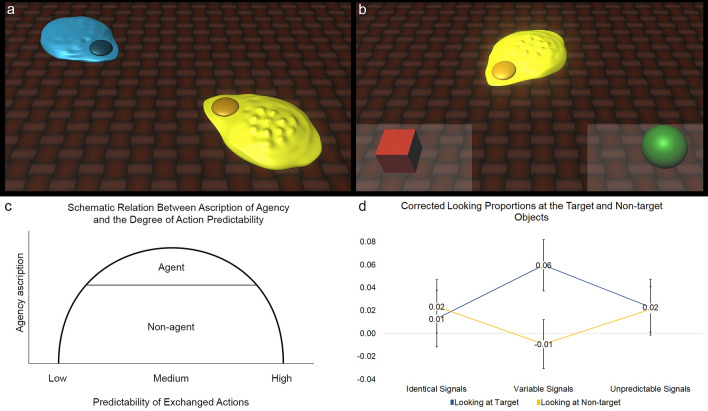
(**a**) Entities exchanging signal sequences in the familiarization phase, (**b**) entity orients toward the target referent in the test phase (light grey areas indicate ROIs around the objects), (**c**) hypothesized relation between predictability of exchanged actions and agency ascription, (**d**) baseline-corrected and log-transformed looking time proportions at the target and non-target object in the three conditions.

## Results

Looking proportions at the target object showed a significant quadratic trend, (*F*(1, 141) = 8.171, *p* = 0.005, 95% CI = [0.018, 0.046]) and medium effect size (*f* = 0.24) as revealed by a one-way ANOVA. This indicates that infants looked more at the target object when the level of predictability in the compositional structure of signal sequences was mid-high (Variable Signals condition: *M* = 0.060, 95% CI = [0.037, 0.083]) in contrast to perfect predictability (Identical Signals condition: *M* = 0.013, CI = [− 0.012, 0.038]) or full unpredictability (*M* = 0.023, 95% CI = [− 0.002, 0.048], see Fig. 1d) of the compositional structure. Looking proportions at the non-target object also showed a significant quadratic trend, (*F*(1, 141) = 5.643, *p* = 0.021, 95% CI = [− 0.001, 0.024]) and small effect size (*f* = 0.20), but in the opposite direction. Infants looked less at the non-target object when the degree of predictability of the contingent signal sequences was mid-high (Variable Signals condition: *M* = − 0.009, 95% CI = [− 0.031, 0.013]) as opposed to perfectly predictable (Identical Signals condition: *M* = 0.024, 95% CI = [0.000, 0.048]) or fully unpredictable (*M* = 0.021, 95% CI = [0.001, 0.041], see Fig. 1d). There was no significant quadratic or linear effect of Condition in looking at the ROI of the Entity (*F*(1, 141) = 0.318, *p* = 0.574, 95% CI = [− 0.104, − 0.062]).

A 3 × 2 repeated measure ANOVA comparing all conditions revealed that the Condition × Object interaction was significant (*F*(2, 138) = 5.401, *p* = 0.006, partial *η*^*2*^ = 0.073, 95% CI = [0.014, 0.030]) indicating that infants looked more at the target object in contrast to the non-target referent in the Variable Signals condition. No other effect was significant when all the three conditions were included in the analysis. To analyze the significant Condition × Object interaction we directly compared the Identical and Variable Signals conditions. The repeated measures ANOVA revealed a significant Condition × Object interaction (F(1, 92) = 9.113, p = 0.003) and significant main effect of Object (F(1, 92) = 4.845, p = 0.03). Infants looked significantly longer at the target compared to the non-target object in the Variable signals condition (t(47) = 3.816, p < 0.001) than in the Identical Signals condition where this difference was not significant (t(47) = 0.566, p = 0.574) and due to this difference they looked longer at the target than the non-target object in the Variable Signals condition. A further repeated measures ANOVA revealed that when comparing the Variable Signals and Unpredictable Signals conditions there was a significant Condition × Object interaction (*F*(1, 92) = 7.004, *p* = 0.01, partial *η*^*2*^ = 0.07, 95% CI = [0.014, 0.033]). Infants’ looking proportions at the target was significantly bigger than at the non-target object in the Variable Signals condition (see above), however, there was no significant difference between looking at the target versus the non-target object in the Unpredictable Signals condition. At the same time there was a significant main effect of Object (*F*(1, 92) = 7.826, *p* = 0.006, partial *η*^*2*^ = 0.08, CI = [0.014, 0.033]) indicating that infants in the present experiment looked more at the target object than at the non-target object on average, presumably due to the orientation-following gaze-response in the Variable Signals condition. A third repeated measures ANOVA was conducted to compare the Identical and Unpredictable Signals conditions directly and revealed no significant main effects or interactions.

## Discussion

The present results suggest that infants are sensitive to the differences in the degree of co-dependent predictability of signal sequences that are exchanged in contingent interactions. We assumed that goal-directed social interactions are in general characterized by partial predictability and the co-dependence of actions performed by cooperative social agents. In line with this assumption 10-month-olds were able to differentiate the partially predictable and co-dependent interactive responses from fully predictable and non-predictable signal exchanges. Moreover—and in accord with our assumption—partial predictability of the exchanged signal sequences proved to be an indicative cue that induced attribution of intentional agency and social interactions by infants, while fully predictable or non-predictable random signal exchanges did not result in agency attribution.

In previous gaze-following experiments of agency attribution when a contingently reactive entity performed a turning action towards one of two lateral target objects it elicited an orientation-following gaze response in the infants to look at the same referent object that the entity’s orienting reaction indicated. In the present paradigm, infants’ orientation following gaze-responses were only induced when the signal sequences exchanged were temporally contingent and co-dependent at the same time. When the consecutive signal sequences of a turn-taking exchange were completely independent and unpredictable or, alternatively, they consisted of fully predictable, persistently repeated identical sound triplets, infants were not induced to perform orientation following gaze responses and so they did not look longer at the target object as compared to the non-target object.

These results significantly extend our understanding of agency attribution in preverbal infants. It seems that apart from detecting contingent reactivity of an entity, 10-month-olds are also sensitive to and rely on the abstract structural cue of partial predictability and co-dependence of the exchanged responses in order to attribute agency to those entities whose behavior is being influenced by and coordinated with the preceding actions of its interacting partner. Importantly, when the predictability of exchanged signals was perfect, the degree of co-determination was too high to enable efficient coordination of interactions to achieve a social goal (irrespective of whether it was perceived as an instrumental shared goal or a communicative goal of exchanging relevant and new information). Therefore, the repetitive exchange of self-induced, but identical behaviors by the turn-taking entities did not induce agency attribution in 10-month-olds. Similarly, when the exchanged signal sequences were randomly variable and lacked co-dependence and predictability, agency attribution did not occur either. This suggests that the fully unpredictable interactions were represented by preverbal infants as not being co-determined, thus lacking an essential property of goal-directed social interactions. Therefore, the present findings suggest that in the domain of social interactions infants’ ascription of agency to the interacting entities follows a non-linear, inverted U-shaped-like function when it is plotted against the degree of unpredictability of signal sequences in interactive exchanges.

Previous findings revealed that infants can rely on the degree of predictability of relevant stimuli in other cognitive domains as well. For instance, infants’ attention and the predictability of the visual stimulus attended follows an inverted U-shape like curve^24^. Accordingly, infants look longer at events (e.g., the appearance of an object from behind an occluder) when it can be characterized by medium–high predictability as opposed to very high or low predictability. This may serve to induce preference towards and longer observation of visual stimuli that maximize the chance of learning^25^ as moderately predictable in contrast to highly predictable or unpredictable events can provide more new and relevant information for the infant. Another line of studies showed that infants are also able to track the degree of predictability of self-induced contingent reactions. The self is necessarily completely contingent with itself, for instance there is a perfect visual-proprioceptive match between the leg movements of an infant and its image on her retina. In contrast, the contingency between interacting others and the self is high-but-imperfect. Thus, the degree of temporal contingency can aid the differentiation between self and other in infants and facilitate learning about the self versus social others^26^. Such results indicate that sensitivity to and active monitoring of the degree of predictability is a basic competence that is present in infants from early on. This further suggests that monitoring quantitative differences in the input information in different cognitive domains can lead to qualitatively different kind representations of the observed entities.

The present findings indicate that apart from kinds of entities, infants can also recognize and differentiate basic types of dynamic events by relying on the degree of mutual predictability of behaviors. They may identify social and communicative interactions based on the medium–high degree of predictability and co-dependence of the exchanged signals or actions and differentiate them from random or repetitive events. We conjecture that this ability can be crucial in human infants’ social and communicative development. It can guide them to represent and learn about the rich social world characteristic of the human species and can help them to recognize *when* a social interaction takes place, which may reveal *which* actions and behaviors can be of social importance and might be relevant to learn about. Thus, it seems that preverbal infants can exploit their early sensitivity to determine the degree of predictability and co-dependence of behavioral exchanges to identify both intentional agents and their goal-directed social interactions, which can be fundamental in early cognitive development.

## Methods

### Subjects

One hundred forty-four, full term, healthy infants were assigned equally to one of six subgroups [3 Conditions (Identical Signals, Variable Signals, Unpredictable Signals) × 2 Signal types (melodic tones, Morse codes)] in a pseudorandom manner (*n* = 144, 85 girls). The infants in the Identical Signals and Variable Signals conditions were tested in a previous study^16^, while the infants in the Unpredictable Signals condition were tested in the present study, therefore the sample size of the Unpredictable Signals condition was matched to the size of the previous samples (*n* = 48, 20 girls). The mean age of infants was 319.31 days (*SD* = 8.19). An additional 31 infants (Identical Signals: 11; Variable Signals: 10; Unpredictable Signals: 10) were excluded due to fussiness^6^, technical error^4^, parental intervention^4^, or because the eye-tracker did not record data in the test phase^17^. Infants were recruited through local birth records. Parents gave their informed consent for their children to participate in an eye tracking study during which video recordings are also made for further data analysis. The infants were given a small toy gift for their participation after the experiment. Ethical approval for the study was obtained from the United Ethical Review Committee for Research in Psychology (EPKEB) in Hungary and it was conducted according to the ethical rules and standards regarding psychological experimentation in Hungary.

### Apparatus

The stimuli were presented by PsyScope X (http://psy.ck.sissa.it) running on a Mac Mini. Eye-tracking data was collected by Tobii T60 XL machine (Stockholm, Sweden) and it was recorded by Tobii Studio 3.4.8 running on a Dell Precision T5820.

### Procedure

The procedure was exactly the same as in Tauzin and Gergely^16^. Before the experiment, a concise description of the study was given to the parents. Infants sat on their parent’s lap during the session, approximately 60 cm away from the display of the eye-tracker. Infants were held in the same position by the parent without turning back the infants’ torso or head if they looked away. Speaking or interacting with the infant was not allowed during the experiment. For later analysis, both the stimuli and the participants were video recorded.

### Stimuli

In all conditions, infants watched 3 familiarization and 6 test videos, which were separated by short attention-getters. The animations in the familiarization phase were 24 s long. The test videos lasted for 4.75 s. If the infant looked away, the attention-getter video was presented until the infant looked back at the screen. When the infant fixated the monitor again the presentation of the attention-getter was stopped by the experimenter and the next animated video was started automatically.

#### Familiarization phase: variable signals condition

Infants were presented with short, animated videos showing a blue and a yellow unfamiliar flat drop-shaped entity in the bottom right and top left corner of the screen orienting towards each other with their pointy ends (see Fig. 1a). The two entities produced signal sequences consisting of three sound units in a turn-taking manner, composed either of Morse codes or melodic tones. Each video showed four turn-taking exchanges of sound triplets. Each of the entities emitted glowing lights emanating from its body simultaneously with the units of the sound sequence to indicate which entity produced the given sound units. Importantly, both entities remained stationary throughout the familiarization phase, they did not produce any agency cues involving self-induced movements or movement-based cues of intentional agency.

In the Variable Signals condition, on average 50% of the exchanged sound units were the same and appeared in the same serial position resulting in a mid-high predictability of signal sequences. The first signal of each sound triplet was the same in each turn in a given familiarization video. The second signal unit was repeated only 50% of the time in the next turn by the subsequent entity. The third unit of the sound triplets was never repeated. For example, after the first entity produced an ‘A–B–C’ signal sequence, the second entity could emit either an ‘A–B–E’ or an ‘A–D–E’ sound triplet as a response. Therefore, the signal sequences in the Variable Signals condition were partially predictable in terms of sound-token identity as well as the serial position occupied by the sound-tokens in the compositional structure of the signal sequence. The first unit of the sound triplets was always novel in each familiarization videos.

The Morse sound code units used were 120, 240, 360 or 480 ms long. The time intervals between ‘dots’ and ‘dashes’ in a Morse code was always 60 ms long. The total length of the Morse code sound triplets varied between 1120 and 1480 ms. The onset of Morse code units was fixed, accordingly, the second unit followed the onset of the first by 500 ms, while the third one always followed the onset of the first by 1000 ms. Each melodic tone was 500 ms long, and their onsets were the same as those of the Morse code units. The interval between the sound triplets produced by the different entities was 1500 ms.

#### Familiarization phase: identical signals condition

In the Identical Signals condition, the predictability of signal sequences exchanged was perfect. The signal triplets produced by the first entity were always repeated exactly by the second entity, and then the same signal triplet was again repeated now by the first entity in a turn-taking manner in a given familiarization video. For instance, the sequence ‘A–B–C’ produced by the first entity was followed by the very same sequence ‘A–B–C’ emitted by the second entity and so on. In each familiarization videos new signal sequences were presented (for example, ‘D–E–F’ in the second video, and ‘G–H–I’ in the third).

#### Familiarization phase: unpredictable signals condition

In the Unpredictable Signals condition, the signal sequences produced in a turn-taking manner were completely unpredictable. The sound units of signal triplets emitted by the first entity were never repeated by the second entity but were followed by a completely independent and fully unpredictable signal triplet during each exchange. For example, the sequence ‘A–B–C’ emitted by the first entity was followed by the unpredictable sequence of ‘D–E–F’ produced by the second entity. After that, a completely new signal sequence was produced by the first entity (G–H–I), and so on. Therefore, in all the familiarization videos the compositional structure of the consecutive signal sequences were fully unpredictable. No sound triplets of a previous familiarization sequence were ever repeated later in the familiarization phase.

#### Test phase

In all conditions, the same test videos were used, which were composed of four identical parts. The entity that was presented in the lower part of the screen during familiarization appeared in the middle of the screen during the test phase with its pointy end orienting toward the bottom center part of the display. At the same time two lateral target objects (a green sphere and a red cube of similar size) appeared at the two lower sides on the screen. The entity in the middle remained stationary for 1500 ms. Then, it emitted a glowing light and a short beep simultaneously. Following this, the entity performed a self-induced body movement by turning its orientation 45 degrees so that its front became aligned with one or the other of the two lateral target objects (see Fig. 1b). After the orientation change, the entity remained still, its front being directed towards one of the target objects for 2000 ms. This test trial was repeated 6 times so that each time the entity oriented towards the same target object, which appeared either on the left or the right side of the screen.

The left–right position of the two lateral objects was counterbalanced across the 6 test trials within infants. The entity appearing in the test phase (yellow or blue), the direction of the first turn it performed, and the specific target object (cube or sphere) toward which it oriented were all counterbalanced between infants.

### Data analysis

In line with Tauzin and Gergely^16^ we measured cumulative looking times distributed across three pre-defined regions of interest (ROI) during the last second of each 6 test videos when the entity was still and oriented towards the target object. Three ROI’s were created by virtually dividing the scene into 3 × 3 equally sized areas first. The ROI of the target and non-target objects were the bottom left and bottom right areas of the scene, while the ROI where the orienting entity itself was positioned included the top center and middle center areas of the scene.

Since the orienting entity remained present throughout the test videos, it was possible that infants’ lack of orientation-following gaze response was the consequence of either looking at the non-target object or looking at the orienting entity itself. Therefore, to avoid disregarding meaningful data by ignoring those trials and time periods when the infant fixated the entity only, we calculated trial-by-trial for each infant the respective proportions of looking at each of the 3 ROIs compared to the overall looking times at all three ROIs together. Trials without any fixation at the ROI of the entity before its turning towards the target object were excluded from subsequent analysis to ensure that the orientation change was noticed by the infants.

We, then, applied baseline-correction on the looking proportions. Since self-propulsion of an entity—for instance, the turning response to align with a target object as performed in the test phase of the present study—can be argued to induce agency attribution in itself^13,27^, the baseline was measured before the orientation change was initiated by the entity in the first test trial, to assess the pre-test distribution of fixations at the three ROIs in the different subgroups. We calculated the average baseline proportion of looking at the ROI of the entity, the target, and the non-target object in each subgroup. This was done to control for the high variability across subjects, which was probably induced by the fact that the stimuli were completely novel for the infants and that there was only one baseline trial per infant. Then, in each subgroup, this average baseline proportion was subtracted from each infants’ corresponding mean looking proportions produced during the test phase. Since the looking proportions at the target and non-target objects were skewed to the left, we applied log-transformation to the baseline-corrected looking time data in accordance with recent methodological recommendations^28^.

We, then, performed one-way ANOVAs with planned quadratic contrast to measure looking proportions at the target and non-target objects as a function of the different levels of predictability of the signal sequences exchanged in the three different conditions (Identical Signals, Variable Signals, Unpredictable Signals). Signal type—as hypothesized—had no significant main effect or interactions with other factors, therefore the participants in the Morse code and melodic signal groups were collapsed when conducting one-way ANOVAs. We also performed repeated measures ANOVAs with Condition and Signal type (Morse code vs. melodic tone) as between subject variables and Object (target vs. non-target) as a within-subject variable to measure the difference in infants’ looking at the lateral objects.
